# Philadelphia Letter

**Published:** 1880-01

**Authors:** 


### ARTICLE VII.

### PHILADELPHIA LETTER.

*Messrs. Editors:*—At the time I write the activity of the medical profession in this city appears to be chiefly displayed in the form of lectures at the colleges and hospitals and in public and private practice. But few papers of general interest have been read before our various societies during the autumn and not many articles have been published in our local journals of such a character as to deserve special mention. It may be supposed that the energies of our working men have been expended in the hospital ward, the laboratory and the studies, and that the fruit of their labors is to appear later in the season. Among the papers of most general interest which have been read at our societies during the past six months, are two which are worthy of notice. The first of these was a paper by Dr. Albert H. Smith, one of our best known gynæcologists, on "The Uses of the Hot Water Douche in Parturition," read before the County Medical Society. Dr. Smith is inclined to the belief that hot water possesses the power of absolutely controlling hæmorrhage from small open vessels and from oozing surfaces in cavities where continuous irrigation can be kept up with the temperature of the water maintained at 110° to 115°, and this without aid from the muscular contractility of the tissues. In order to obtain the benefit of the hot douche in hæmorrhage in pregnancy, the free and unobstructed play of the muscular contractility of the uterus is first required and next, especially, free access of the stream directly to the bleeding surface. The uterus must first be emptied. No special apparatus is required; an ordinary Davidson's syringe, a fountain or bag syringe, or even a rubber tube, which every country practitioner should carry with him for many useful purposes, may be employed.

The hot water douche may be employed (1) to relax a rigid os and hasten the first stage of labor. (2.) To produce prompt and vigorous condensation of the uterine walls after expulsion of the fœtus. (3.) To arrest hæmorrhage from laceration in the passage of the child through the pelvic canal. I wish I could give a further account of this paper, one of the most practically valuable which has been brought out by the general interest in hot water as a hæmostatic that has prevailed during the past year. Any of your readers who, not having seen Dr. Smith's paper, would like to read it in full will find it in the *Philadelphia Medical Times*, Vol. IX, 1879, p. 541.

Scarcely of less interest is the paper of Dr. John B. Roberts, read before the same society, on "Paracentesis of the Pericardium." Dr. Roberts considers this operation not only advisable but necessary in many cases of cardiac trouble accompanied by effusion and as not at all difficult to perform. His paper has called forth much comment and he has been supported in his views by Professor Pepper in a paper published in the October number of the *American Journal of the Medical Sciences.* Within a week or two Dr. Roberts has published a monograph with the same title, giving the results of the latest experience in this operation.

Although the journals have not recently teemed with papers by Philadelphia men, yet this has not been an idle year and the number of new works of value and of new editions of old works has not been small. Agnew's great work on surgery, the first volume of which appeared near the close of last year, is approaching completion and in wealth of information, particularly of a statistical sort, is without a peer in works of its kind, though in beauty of style and literary arrangement it is not equal to others. Professor Ashurst, who is Professor Agnew's colleague in the university, occupying the chair of clinical surgery has just brought out the second edition of his "Principles of Surgery." Professor Harrison Allen has long been engaged on a work on anatomy, of which Dr. Shakspeare has written the histological introduction. This *opus magnum* is at length to see the light, as I believe it is to appear early in the year. Those who know Professor Allen's painstaking, laborious and thorough method, expect the appearance of this work with great interest, and confidently hope for a volume which will add to the credit of American medical science in a field hitherto left uncultivated.

Professor Duhring's "Treatise on Diseases of the Skin," which has for some time been out of print, is about to appear in a second edition, revised and brought up to the most recent standard of knowledge of these affections, and of course necessarily enlarged. Duhring's "Atlas of Skin Diseases," a production of permanent value, in which the profession of this city feel a certain pride, not unwarranted by the character of the work, continues to appear in parts from time to time, and is, I believe, a little more than half way to completion. In a very different class must be reckoned the series of "Health Primers," of which various volumes, under the editorship of Dr. Keen, and chiefly written by Philadelphia men, have been making their appearance during the last six months. Although these works are written in a popular style, they contain much of interest and instruction for the medical man, and are not to be tossed lightly aside as unworthy of serious notice. In this respect they compare favorably with the primers of a similar sort issued in England, one of which lies before me as I write, which it would not be too much to denominate trashy. Some of Dr. Keen's "Health Primers" have been written by specialists of standing, and those of Dr. Burnett on the ear, Dr. Harlan on the eye, and Dr. J. W. White on the teeth, may be confidently recommended as containing just the sort of information on these special organs which specialists alone can give, and which is hardest to obtain without going more deeply into these subjects than the average doctor's time will allow. We of the profession must be alive to these points, or we are apt to be caught up and our ignorance exposed by clever laymen who have gained the latest fruit of modern science from these little manuals. I dare say, however, that many of your readers have seen the "Health Primers," for their sale has been enormous, and the publishers, I fancy, have reason to bless the day when their enterprise was undertaken.

One more Philadelphia book of recent date may be mentioned which has gained great success—that is, Stillé and Maisch's "National Dispensatory," of which the second edition is rapidly passing off, although scarcely half a year has elapsed since its first publication. To one of its authors, Professor Stillé, this work is peculiarly honorable, because it has been undertaken and carried through at an age when most men have laid aside their more laborious occupations and are beginning to enter into the rest which they have earned by their labors, and to enjoy whatever laurels they may have won. It is now about a year, I think, since Professor Stillé handed in his resignation to the trustees of the University of Pennsylvania, and was about to retire from the Chair of Practice which he has filled so well for the last seventeen years, when he was induced, by the strenuous efforts of the students and alumni, together with the friends of the institution, to remain during a somewhat longer period. The reason assigned for Professor Stillé's resignation of his important position, when to all appearance he was at the height of his usefulness, seems to have been a certain resolution, made many years ago, to the effect that when he had reached a certain age, sixty-five I think, he would retire, although he might be in the enjoyment of all his faculties. The origin of this self-sacrificing resolution is, I venture to think, to be found in a certain yellow and discolored pamphlet, dating somewhere about 1832, and containing a vigorous protest against the retention of the Chair of Materia Medica in the university by the then occupant of that position. This protest was signed by a committee of students, at the head of which appears the name of Alfred Stillé. It sets forth in forcible language the grievances of which the unfortunate occupants of the benches were the sufferers, stating that although the subject which Professor C—— was supposed to teach was materia medica, yet a number of his lectures were occupied with a discussion of Galen's views on the anatomy of man, as derived from dissections of monkeys. Moreover, when at last the true subject of the lectures was reached, no allusions were made to writers of a later date than Dioscorides and Avicenna, as the venerable professor considered all discoveries of a later date as vain, trifling and of little account. Moreover, it was added, if my recollection serves me, that age had so far impaired the utterance of the professor that but little of what he said could be heard even by the acutest ear. However, as the chair was at that time worth some $10,000 a year to its holder, the superannuated occupant was removed with difficulty, and I suppose that the memory of that student experience caused Professor Stillé to resolve not to dim the luster of his reputation by lagging superfluous on the stage. That he is far from even the beginning of a decline of his powers is seen in the work which he has just completed, to which allusion has been made, and that he may long be spared to adorn his chair is the fervent hope of all friends of the university. The Jefferson College has sustained a severe loss in the death of Dr. J. Aitkin Meigs, professor of the Institutes of Medicine. Professor Meigs was known to the scientific world generally rather as an ethnologist than as a physiologist, having written little or nothing upon the latter subject. As a lecturer he was deservedly popular, on account of the earnest eloquence of his style and the abundant illustration which he introduced into his lectures. He was the first lecturer in this city, and one of the first in the country, to introduce vivisectional illustrations. Although bearing the same name, yet he was in no way related to the other physicians of the Meigs family who have practiced in Philadelphia for several generations. It appears, by the way, to be one of our characteristics to produce successive generations of medical men of ability. Charles D. Meigs was the famous obstetrician and rival of Hodge of fifty years ago. John F. Meigs, his son, is the joint author with Professer Pepper of the well-known "Meigs and Pepper on Diseases of Children;" while a grandson of the family is a well-known practitioner.

A new society has recently sprung into existence, called the "Academy of Surgery." Professor Gross is believed to be the originator of this association, which is to be very select, only numbering some thirty members. I hope to be able to inform your readers further regarding this and our other societies at some future date. PHILA.

Dec. 17, 1879.

